# Cumulative Score Based on Preoperative Fibrinogen and Pre-albumin Could Predict Long-term Survival for Patients with Resectable Gastric Cancer

**DOI:** 10.7150/jca.35157

**Published:** 2019-10-17

**Authors:** Zhi-jun Wu, Hui Xu, Rong Wang, Li-jia Bu, Jie Ning, Ji-qing Hao, Guo-ping Sun, Tai Ma

**Affiliations:** 1 Department of Oncology, the First Affiliated Hospital of Anhui Medical University, Hefei, Anhui, 230022, P. R. China.; 2 Department of Oncology, Maanshan People's Hospital, Maanshan, Anhui, 243000, P. R. China.; 3 Anhui Institute for Cancer Prevention and Control, Hefei, Anhui, 230022, P. R. China.; Zhi-jun Wu and Hui Xu contributed equally to this work.

**Keywords:** Gastric cancer, Gastrectomy, Fibrinogen, Pre-albumin, Survival

## Abstract

**Background**: To investigate the prognostic significance of the cumulative score based on preoperative fibrinogen and pre-albumin (FP score) in patients with gastric cancer after radical gastrectomy.

**Methods**: Baseline characteristics, preoperative fibrinogen and pre-albumin levels were retrospectively reviewed in patients who underwent radical gastrectomy. The optimal cut-off values for fibrinogen and pre-albumin were defined as 4.0 g/L and 230.0 mg/L, respectively. Patients with elevated fibrinogen (≥ 4.0 g/L) and decreased pre-albumin (< 230.0 mg/L) levels were allocated an FP score of 2, those with only one of these two abnormalities were assigned a score of 1, and those with neither of the two abnormalities were allocated a score of 0. The prognostic value was examined by univariate and multivariate regression analyses.

**Results**: The preoperative FP score was significantly correlated with age, tumor size, fibrinogen level, pre-albumin level and white blood cell count. No significant differences based on sex, tumor location, degree of differentiation, depth of invasion, lymph node status, tumor-node-metastasis (TNM) stage or adjuvant chemotherapy were identified between the groups. In addition, univariate survival analysis revealed that a high preoperative FP score was significantly associated with unfavorable disease-free survival (DFS) [hazard ratio (HR), 1.482; 95% confidence interval (CI), 1.222-1.796; *P* < 0.001] and overall survival (OS) (HR, 1.623; 95% CI, 1.315-2.002; *P* < 0.001). Moreover, after adjusting for other factors, a high preoperative FP score remained an independent predictor for impaired DFS (HR, 1.434; 95% CI, 1.177-1.747; *P* < 0.001) and OS (HR, 1.413; 95% CI, 1.136-1.758; *P* = 0.002) in multivariate Cox regression analysis.

**Conclusions**: The preoperative FP score significantly predicts long-term survival for gastric cancer patients who have undergone radical gastrectomy.

## Introduction

Gastric cancer is one of the deadliest malignancies worldwide and remains the second most common cancer and the second leading cause of cancer-related death in China 1, 2. Surgery is the only curative approach for resectable cases, either alone or in combination with adjuvant treatment 3. However, most patients present with advanced disease at initial diagnosis, thus missing the chance to undergo radical resection. In addition, the rates of recurrence and distant metastasis in subjects undergoing radical gastrectomy remain high 3. Moreover, although great advances have been made in the early screening, diagnosis and treatment of gastric cancer, the prognosis remains poor, with an estimated 5-year overall survival (OS) rate of less than 40% 2, 3.

Various tumor related-factors, including tumor size, degree of differentiation, depth of invasion, lymph node status and distant metastasis, along with patient-related factors such as age, sex, and comorbidity have been identified as important prognostic indicators for gastric cancer patients 4. In addition, some inflammation-based prognostic variables such as the platelet-lymphocyte ratio (PLR), neutrophil-lymphocyte ratio (NLR) and lymphocyte-monocyte ratio (LMR) have also been established as predictors of long-term survival in such cases 5-7. However, more accurate and promising biological markers are still needed to classify the risk of unfavorable prognosis and to design optimal therapeutic strategies for patients with gastric cancer.

Fibrinogen, a glycoprotein produced by hepatic cells, is a key regulator of the hemostatic system and plays important roles in blood coagulation, cell-cell adhesion and the systemic inflammatory response 8. In addition, elevated fibrinogen levels have been observed in various malignancies, including gastric cancer, and could promote tumor progression, invasion and distant metastasis 9. Moreover, hyperfibrinogenemia has also been confirmed to be significantly correlated with increased tumor size, advanced tumor stage, and poor prognosis in gastric cancer patients 10-14. Furthermore, decreased pre-albumin levels are also frequently observed in gastric cancer patients and are correlated with unfavorable survival 15. Most recently, Zhang and his colleagues suggested that the preoperative fibrinogen/pre-albumin ratio (FPR) might be a novel prognostic indicator in patients with surgical stage II and III gastric cancer and that it could precisely distinguish stage III patients who would benefit from adjuvant chemotherapy 16.

Therefore, we proposed that a cumulative score based on preoperative fibrinogen in combination with pre-albumin (FP score) might provide more accuracy in predicting long-term survival for resectable gastric cancer patients. The purpose of this study was to examine the correlation of the preoperative FP score with clinicopathologic variables, and to investigate its prognostic significance in resectable gastric cancer patients.

## Methods

### Patients

The electronic medical records of 396 patients with newly diagnosed gastric cancer from April 2007 to August 2016 in the First Affiliated Hospital of Anhui Medical University in Hefei, China were retrospectively reviewed. Only patients who underwent radical gastrectomy and had histopathologically confirmed gastric cancer were enrolled in the present study. Patients who were diagnosed with other malignancies, underwent neoadjuvant chemotherapy and/or radiotherapy, or had diseases needing anticoagulants that would affect the hemostatic system were excluded. In addition, we excluded subjects without preoperative information on nutrition and hemostasis and patients who died of causes other than gastric cancer. Furthermore, those diagnosed with chronic inflammatory diseases, malnutrition or infections were also excluded. Therefore, a total of 306 cases were enrolled in the final analysis.

### Treatment and follow-up

All included patients underwent radical gastrectomy. The median number of dissected lymph nodes was 17 (range, 2 to 68). A total of 243 patients with high rate of local recurrence and/or distant metastasis received adjuvant chemotherapy. Fluorouracil-based two-drug combination chemotherapy was delivered to four-fifths of the patients, whereas the remaining subjects underwent fluorouracil monotherapy. Regular blood tests, including the detection of tumor markers, ultrasound/computed tomography and upper gastrointestinal endoscopy were regularly evaluated after surgery. The patients were followed up via the telephone. The time from resection to recurrence and metastasis recorded by imaging (CT, B-mode ultrasound, MRI, etc.) or histopathological cytology or the time from resection to the last date of follow-up was defined as the DFS. OS was calculated from the date of resection to death from cancer or the most recent follow-up.

### Clinical and laboratory variables

The patients' baseline characteristics, preoperative fibrinogen and pre-albumin levels, and other parameters, were retrieved and collected from the electronic medical records. Tumor stages were classified according to the AJCC/UICC TNM staging system (the 7th edition). The long diameter measured on the general post-operative pathological specimen was considered the tumor size. The tumor locations were divided into upper, middle, lower and diffuse stomach. The degree of differentiation was categorized into poorly/not differentiated and moderately/well differentiated. The preoperative fibrinogen and pre-albumin concentrations were determined in samples collected within one week before surgery. Plasma fibrinogen levels were tested by an automatic coagulation analyzer (CS-5100, Sysmex, Japan). Serum pre-albumin levels were examined using an automatic biochemical analyzer (Cobas 8000, Roche, Switzerland).

### Fibrinogen and pre-albumin score (FP score)

The optimal cut-off value for preoperative fibrinogen was defined as 4.0 g/L according to previous studies and the cut-off value for pre-albumin was determined as 230.0 mg/L with the method available in the X-tile 3.6.1 software 17, 18 (Yale University, New Haven, CT, USA). Patients with elevated fibrinogen (≥ 4.0 g/L) and decreased pre-albumin (< 230.0 mg/L) levels were allocated an FP score of 2, those with only one of these two abnormalities were assigned a score of 1, and those with neither of the two abnormalities were allocated a score of 0.

### Statistical analysis

A chi-square test was used to examine the differences between groups. Survival curves were calculated by the Kaplan-Meier method, and differences were compared with the log-rank test. Cox proportional hazards regression models were utilized to perform univariate and multivariate analyses, and hazard ratios (HRs) for parameters related to DFS and OS were calculated. HRs with 95% confidence intervals (CIs) and two-sided *P* values were reported. All statistical analyses were performed with SPSS 22.0 (SPSS Inc., Chicago, IL, USA). A two-sided *P* < 0.05 was considered statistically significant.

## Results

### Patient characteristics

The baseline characteristics are summarized in Table 1. The median age at diagnosis was 60.0 years (range, 21.0-86.0 years). Nearly two-thirds (69.3%) of the patients were males. Most of them (84.0%) presented with T3/T4 disease. Lymph node metastasis was positive in 244 (79.7%) of the patients. Of these, 26 (8.5%) had stage I, 60 (19.6%) had stage II and 220 (71.9%) had stage III. Four-fifths (243, 79.4%) of the cases received adjuvant chemotherapy (Table 1).

### Correlation of preoperative FP score with clinicopathologic variables

**Figure 1:**
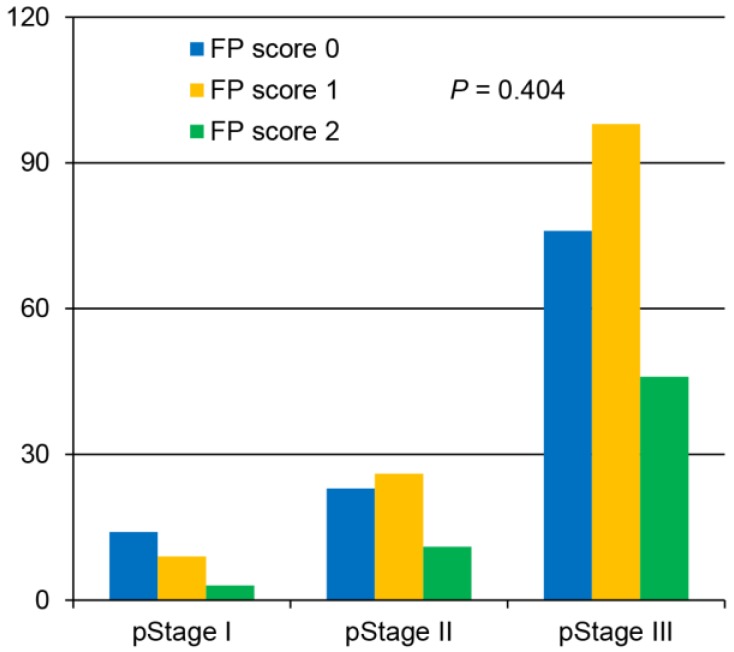
Correlation between the preoperative fibrinogen and pre-albumin (FP) score and the postoperative pathological stage.

Of the 306 enrolled patients, 113 (36.9%) were assigned an FP score of 0, 133 (43.5%) had an FP score of 1, and 60 (19.6%) had a score of 2 (Table 1). The analysis demonstrated that the preoperative FP score was significantly correlated with age, tumor size, fibrinogen level, pre-albumin level and white blood cell count. However, no significant differences based on sex, tumor location, degree of differentiation, depth of invasion, lymph node status, tumor-node-metastasis (TNM) stage or adjuvant chemotherapy were identified among the groups (Table 1, Figure 1).

### Prognostic significance of preoperative FP score in resectable gastric cancer

**Figure 2:**
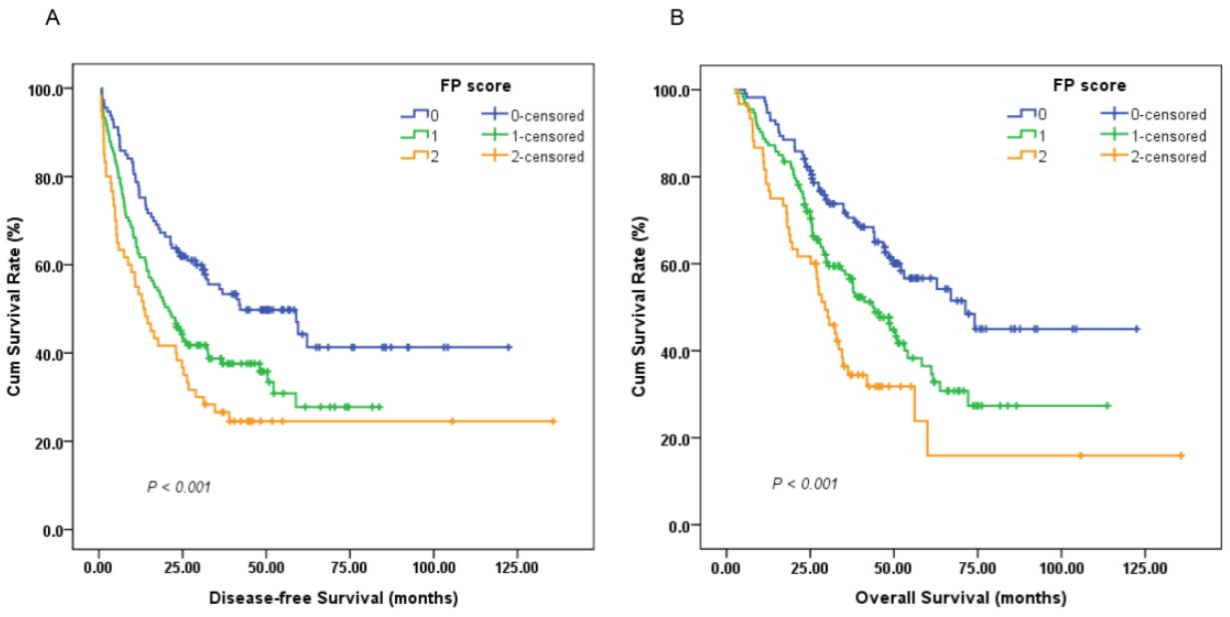
Kaplan-Meier survival curves of (A), disease-free survival (DFS) and (B), overall survival (OS) stratified by preoperative FP score in 306 resectable gastric cancer (GC) patients (log-rank test).

A survival analysis was then performed to evaluate the prognostic value of the preoperative FP score. A Cox univariate model for DFS revealed that a high preoperative FP score was significantly associated with impaired DFS (HR, 1.482; 95%CI, 1.222-1.796; *P* < 0.001; Figure 2A). Tumor size (<5/≥5 cm), depth of invasion (T1-2/T3-4), lymph node involvement (negative/positive), TNM stage (I-II/III), adjuvant chemotherapy (yes/no), fibrinogen level (<4.0/≥4.0 g/L) and pre-albumin level (<230.0/≥230.0 mg/L) were other significant prognostic parameters identified by univariate analysis (*P* < 0.05). In the multivariate analysis, the preoperative FP score (HR, 1.434; 95% CI, 1.177-1.747; *P* < 0.001) remained an independent prognostic indicator for DFS. TNM stage (HR, 2.464; 95% CI, 1.810-3.356; *P* < 0.001) and adjuvant chemotherapy (HR, 0.213; 95% CI, 0.154-0.294; *P* < 0.001) were other independent prognostic factors (Table 2).

Univariate analysis of OS indicated that patients with high preoperative FP scores tended to have unfavorable OS (HR, 1.623; 95% CI, 1.315-2.002; *P* < 0.001; Figure 2B). In addition, other parameters, including age (<60/≥60 years), tumor size, depth of invasion, lymph node involvement, TNM stage, adjuvant chemotherapy, and fibrinogen and pre-albumin levels, could also significantly predict OS. Multivariate analysis was then performed with a Cox proportional hazards model. After adjusting for other confounding variables, we found that a high preoperative FP score could also serve as an independent predictor for OS (HR, 1.413; 95% CI, 1.136-1.758; *P* = 0.002). As expected, TNM stage (HR, 2.812; 95% CI, 1.941-4.075; *P* < 0.001) and adjuvant chemotherapy (HR, 0.382; 95% CI, 0.272-0.538; *P* < 0.001) were two additional significant predictors of OS (Table 3).

**Figure 3:**
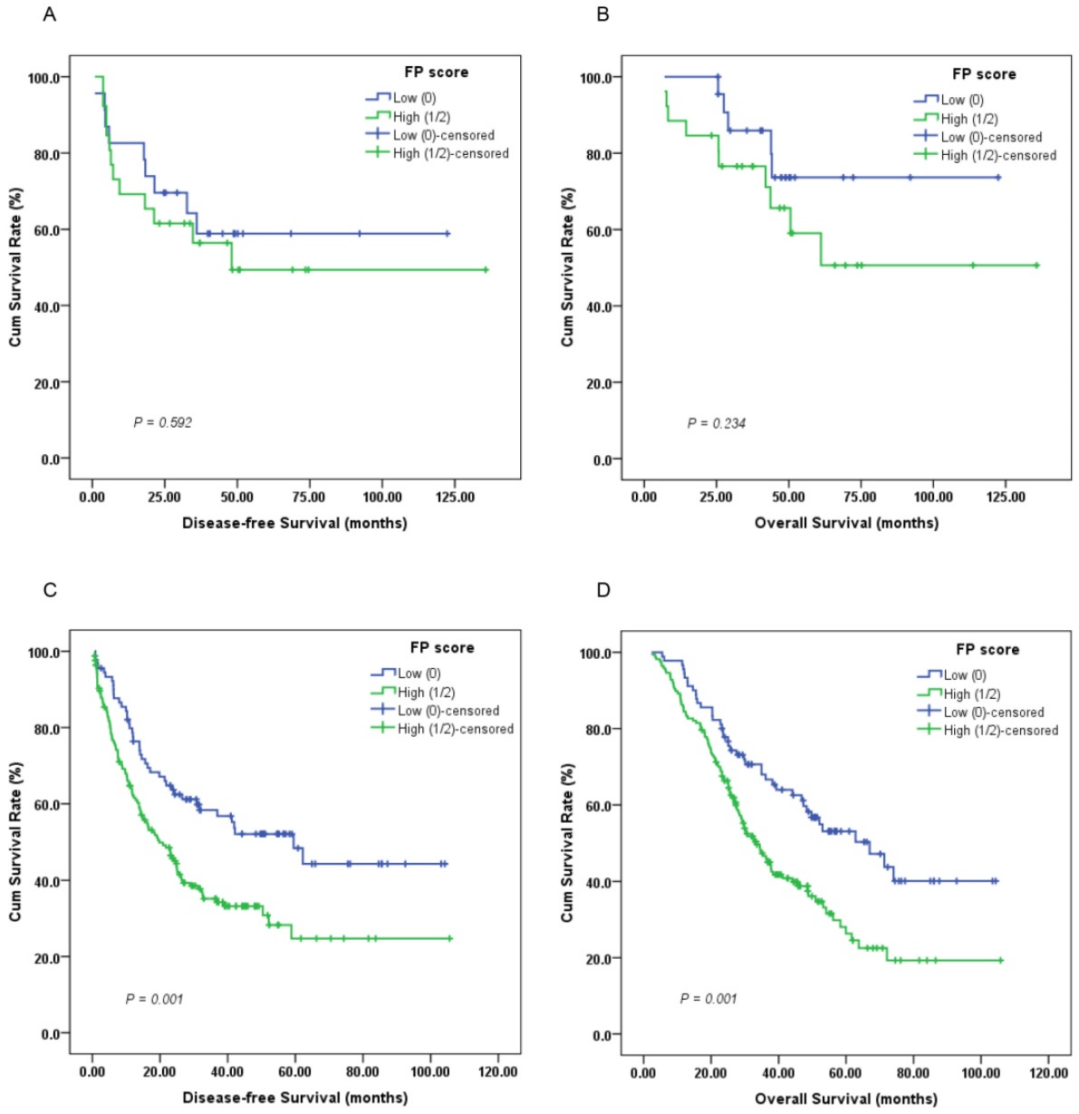
Kaplan-Meier survival curves of (A), DFS and (B), OS stratified by preoperative FP score in T1-2 stage GC patients (N = 49); (C), DFS and (D), OS stratified by preoperative FP score in T3-4 stage GC patients (N = 257) (log-rank test).

**Figure 4:**
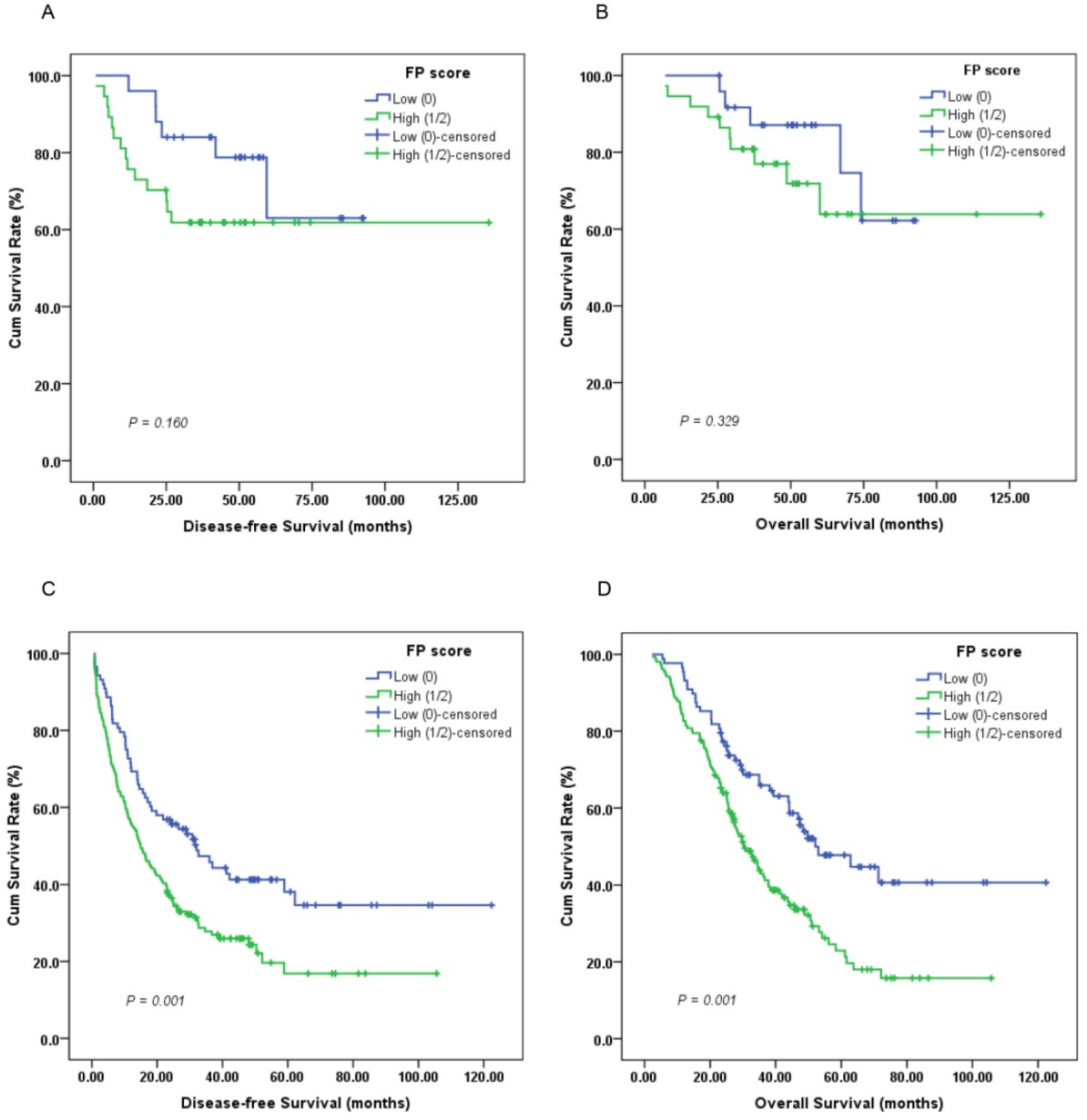
Kaplan-Meier survival curves of (A), DFS and (B), OS stratified by preoperative FP score in GC patients without lymph node involvement (N = 62); (C), DFS and (D), OS stratified by preoperative FP score in GC patients with lymph node involvement (N = 244) (log-rank test).

Furthermore, subgroup analysis indicated that a high preoperative FP score was significantly correlated with unfavorable DFS (Figure 3C, 4C; *P* < 0.05) and OS (Figure 3D, 4D; *P* < 0.05) in patients with T3-4 and lymph node positive disease but not DFS or OS in those with T1-2 (Figure 3A-B; *P* > 0.05) or lymph node negative disease (Figure 4A-B; *P* > 0.05).

## Discussion

To the best of our knowledge, this study was the first to investigate the prognostic significance of the preoperative FP score in resectable gastric cancer patients. The results showed that the preoperative FP score was significantly correlated with systematic inflammation and the clinical outcome, indicating that those with high preoperative FP scores had a relatively higher risk of local recurrence or distant metastasis, as well as worse prognosis. Therefore, intensive neoadjuvant or adjuvant treatment is strongly suggested for such patients. In addition, subgroup analysis revealed that a high preoperative FP score could significantly predict unfavorable survival in cases with more advanced disease.

It has been recognized that the systemic inflammation response and nutrition status are significantly correlated with tumor progression and prognosis in various malignancies, including gastric cancer 19-22. Researchers have found that anti-inflammatorg treatment and perioperative nutritional support could reduce the susceptibility to gastric cancer, prevent disease progression and improve the clinical outcome 22, 23. In addition, as two crucial inflammatory and nutritional markers, elevated plasma fibrinogen and decreased serum pre-albumin levels have been frequently observed in gastric cancer patients and are associated with poor survival 10-15. Suzuki T and his colleagues demonstrated that hyperfibrinogenemia was significantly associated with tumor progression and was an independent indicator of poor prognosis (HR, 2.607; 95 % CI, 1.180-5.761; *P* = 0.018) in patients with gastric cancer 14. In addition, Yu X, et al. found that preoperative serum fibrinogen levels were positively correlated with advanced tumor stages and poor survival in gastric cancer subjects undergoing gastrectomy; these markers could also serve as independent risk factors (HR, 1.36; 95 % CI, 1.14-1.62; *P* < 0.001) for survival in these patients 13. Furthermore, rather than serving as a marker of malnutrition, a decreased pre-albumin level has been identified as an inflammatory indicator and is considered a novel and feasible predictor of unfavorable OS in gastric cancer patients 15. Recently, Han WX, et al. suggested that the preoperative pre-albumin level was closely associated with the hemoglobin level, degree of differentiation and TNM stage. Moreover, it was an independent prognostic indicator (HR, 0.512; 95 % CI, 0.282-0.927; *P* = 0.027), and a low level of pre-albumin was correlated with poor survival in patients with adenocarcinoma of the esophagogastric junction (AEG) who underwent gastrectomy 15. Therefore, it was hypothesized that in combination with fibrinogen, pre-albumin might provide more accuracy in predicting long-term survival in gastric cancer patients.

Most recently, Zhang J and his colleagues investigated the prognostic significance of the preoperative fibrinogen/pre-albumin ratio (FPR) in gastric cancer patients undergoing surgery 16. They found that an elevated preoperative FPR was significantly associated with more advanced tumor invasion, lymph node metastasis and larger tumor size, and it was superior to the levles of fibrinogen, albumin and pre-albumin with regard to independently predicting poor survival in such cases. Moreover, among stage III patients, those with a low FPR appeared to more clearly benefit from adjuvant chemotherapy in comparison with those with a high FPR 16. Consistent with their study, we demonstrated in the present study that the preoperative FP score was significantly correlated with age, tumor size, fibrinogen level, pre-albumin level and white blood cell count, and a high preoperative FP score could significantly predict unfavorable DFS and OS. Moreover, it remained an independent predictor of impaired DFS and OS in the multivariate Cox regression analysis. To the best of our knowledge, this study was the first to report the prognostic value of the preoperative FP score, which was established based on the preoperative fibrinogen and pre-albumin levels in resectable gastric cancer patients.

Although the main limitations of this study were the lack of measurement of other inflammation parameters, the retrospective single-center design and the small sample size, the results showed that the preoperative FP score might serve as a novel and promising marker to predict long-term survival, help more accurately classify patients according to their levels of risk and design optimal therapeutic strategies for resectable gastric cancer patients. However, further studies with large cohorts are warranted to validate these findings.

## Figures and Tables

**Table 1:** Patient baseline characteristics and their correlations with the preoperative FP score (N = 306)

Clinicopathologic	Patients	FP score (N, %)			*P*
Characteristics	N (%)	0	1	2	value
**Age (years)**					< 0.001^*^
< 60	143 (46.7)	56 (49.6)	72 (54.1)	15 (25.0)	
≥ 60	163 (53.3)	57 (50.4)	61 (45.9)	45 (75.0)	
**Gender**					0.314
Male	212 (69.3)	84 (74.3)	87 (65.4)	41 (68.3)	
Female	94 (30.7)	29 (25.7)	46 (34.6)	19 (31.7)	
**Tumor location**					0.973
Upper	123 (40.2)	43 (38.1)	55 (41.4)	25 (41.7)	
Middle	60 (19.6)	24 (21.2)	23 (17.3)	13 (21.7)	
Lower	72 (23.5)	27 (23.9)	33 (24.8)	12 (20.0)	
Diffuse	51 (16.7)	19 (16.8)	22 (16.5)	10 (16.6)	
**Tumor size (cm)**					< 0.001^*^
< 5	131 (42.8)	60 (53.1)	53 (39.8)	18 (30.0)	
≥ 5	175 (57.2)	53 (46.9)	80 (60.2)	42 (70.0)	
**Differentiation**					0.694
Well/Moderate	71 (23.2)	29 (25.7)	28 (21.1)	14 (23.3)	
Poor/Undifferentiated	235 (76.8)	84 (74.3)	105 (78.9)	46 (76.7)	
**T stage**					0.193
T1/T2	49 (16.0)	23 (20.4)	20 (15.0)	6 (10.0)	
T3/T4	257 (84.0)	90 (79.6)	113 (85.0)	54 (90.0)	
**Lymph node status**					0.810
Negative	62 (20.3)	25 (22.1)	26 (19.5)	11 (18.3)	
Positive	244 (79.7)	88 (77.9)	107 (80.5)	49 (81.7)	
**TNM stage**					0.404
I	26 (8.5)	14 (12.4)	9 (6.8)	3 (5.0)	
II	60 (19.6)	23 (20.4)	26 (19.5)	11 (18.3)	
III	220 (71.9)	76 (67.2)	98 (73.7)	46 (76.7)	
**Adjuvant chemotherapy**					
No	63 (20.6)	17 (15.0)	28 (21.1)	18 (30.0)	0.067
Yes	243 (79.4)	96 (85.0)	105 (78.9)	42 (70.0)	
**Preoperative fibrinogen level**					< 0.001^*^
[g/L; median (range)]	3.36 (1.19-7.15)	3.00 (1.19-3.97)	3.26 (1.33-5.18)	4.53 (4.01-7.15)	
**Preoperative pre-albumin level**					< 0.001^*^
[g/L; median (range)]	224.5 (77.0-409.0)	268.0 (231.0-409.0)	203.0 (86.0-342.0)	180.0 (77.0-225.0)	
**Preoperative WBC count**					< 0.001^*^
[(k/cm^3^); median (range)]	5.39 (2.47-14.16)	5.24 (2.70-14.16)	5.07 (2.47-10.63)	6.94 (3.51-14.15)	

FP, fibrinogen and pre-albumin; TNM, tumor-node-metastasis; WBC, white blood cell. ^*^P < 0.05.

**Table 2:** Clinicopathological factors, FP score, and DFS: univariate and multivariate analyses (N = 306)

Variables	Univariate analysis			Multivariate analysis		
	HR	95% CI	*P* value	HR	95% CI	*P* value
Age (< 60 vs. ≥ 60 years)	1.216	0.911-1.263	0.183			NI
Gender (Male vs. Female)	1.008	0.740-1.373	0.959			NI
Tumor location (Upper/Middle vs. Lower/Diffuse)	1.027	0.902-1.168	0.689			NI
Tumor size (< 5 vs. ≥ 5 cm)	1.611	1.191-2.178	0.002^*^	1.258	0.920-1.720	0.151
Differentiation (Well/Moderate vs. Poor/Undifferentiated)	1.139	0.803-1.615	0.466			NI
Depth of invasion (T1/T2 vs. T3/T4)	1.820	1.155-2.868	0.010^*^			NI
Lymph node involvement (Negative vs. Positive)	3.073	1.930-4.893	< 0.001^*^			NI
TNM stage (I vs. II/III)	2.189	1.613-2.970	< 0.001^*^	2.464	1.810-3.356	< 0.001^*^
Adjuvant chemotherapy (No vs. Yes)	0.290	0.211-0.397	< 0.001^*^	0.213	0.154-0.294	< 0.001^*^
Pre-albumin (< 230 vs. ≥ 230 g/L)	1.639	1.221-2.200	0.001^*^			NI
Fibrinogen (< 4 vs. ≥ 4 g/L)	1.559	1.149-2.115	0.004^*^			NI
FP score (0 vs. 1/2)	1.482	1.222-1.796	< 0.001^*^	1.434	1.177-1.747	< 0.001^*^

DFS, disease-free survival; HR, hazard ratio; CI, confidence interval; NI, not included. ^*^P < 0.05.

**Table 3:** Clinicopathological factors, FP score, and OS: univariate and multivariate analyses (N = 306)

Variables	Univariate analysis			Multivariate analysis		
	HR	95% CI	*P* value	HR	95% CI	*P* value
Age (< 60 vs. ≥ 60 years)	1.474	1.078-2.016	0.015^*^	1.383	1.002-1.909	0.049^*^
Gender (Male vs. Female)	0.898	0.643-1.255	0.529			NI
Tumor location (Upper/Middle vs. Lower/Diffuse)	1.002	0.873-1.151	0.975			NI
Tumor size (< 5 vs. ≥ 5 cm)	1.728	1.244-2.399	0.001^*^	1.212	0.862-1.704	0.269
Differentiation (Well/Moderate vs.Poor/Undifferentiated)	1.323	0.903-1.940	0.151			NI
Depth of invasion (T1/T2 vs. T3/T4)	2.365	1.390-4.062	0.002^*^			NI
Lymph node involvement (Negative vs. Positive)	3.616	2.123-6.159	< 0.001^*^			NI
TNM stage (I vs. II/III)	2.758	1.919-3.965	< 0.001^*^	2.812	1.941-4.075	< 0.001^*^
Adjuvant chemotherapy (No vs. Yes)	0.415	0.296-0.580	< 0.001^*^	0.382	0.272-0.538	< 0.001^*^
Pre-albumin (< 230 vs. ≥ 230 g/L)	1.853	1.345-2.554	< 0.001^*^			NI
Fibrinogen (< 4 vs. ≥ 4 g/L)	1.670	1.205-2.315	0.002^*^			NI
FP score (0 vs. 1/2)	1.623	1.315-2.002	< 0.001^*^	1.413	1.136-1.758	0.002^*^

OS, overall survival. ^*^P < 0.05.

## Abbreviations

FP: fibrinogen and pre-albumin
GC: gastric cancer
OS: overall survival
DFS: disease-free survival
TNM: tumor-node-metastasis
HR: hazard ratio
CI: confidence interval
WBC: white blood cell
PLR: platelet-lymphocyte ratio
NLR: neutrophil-lymphocyte ratio
LMR: lymphocyte-monocyte ratio
FPR: fibrinogen/pre-albumin ratio
AEG: adenocarcinoma of esophagogastric junction
NI: not included
